# Pan-Genome Analysis of Effectors in Korean Strains of the Soybean Pathogen *Xanthomonas citri* pv. *glycines*

**DOI:** 10.3390/microorganisms9102065

**Published:** 2021-09-30

**Authors:** In-Jeong Kang, Kyung Seok Kim, Gwyn A. Beattie, Jung-Wook Yang, Kee Hoon Sohn, Sunggi Heu, Ingyu Hwang

**Affiliations:** 1 Division of Crop Cultivation and Environment Research, National Institute of Crop Science, Suwon 16613, Korea; hg369732@korea.kr; 2 Department of Agricultural Biotechnology, Seoul National University, Seoul 08826, Korea; ingyu@snu.ac.kr; 3 Department of Natural Resources Ecology and Management, Iowa State University, Ames, IO 50010, USA; kkssky@gmail.com; 4 Department of Plant Pathology and Microbiology, Iowa State University, Ames, IA 50011, USA; gwynbeattie1@gmail.com; 5 Department of Life Sciences, Pohang University of Science and Technology, Pohang 37673, Korea; khsohn@postech.ac.kr; 6 School of Interdisciplinary Bioscience and Bioengineering, Pohang University of Science and Technology, Pohang 37673, Korea; 7 Department of Plant Science, Seoul National University, Seoul 08826, Korea; sunggiheu@snu.ac.kr

**Keywords:** *Xanthomonas citri* pv. *glycines*, whole genome, transcription activator-like effectors, effector-binding elements

## Abstract

*Xanthomonas citri* pv. *glycines* is a major pathogen of soybean in Korea. Here, we analyzed pathogenicity genes based on a comparative genome analysis of five Korean strains and one strain from the United States, 8ra. Whereas all six strains had nearly identical profiles of carbohydrate-active enzymes, they varied in diversity and number of candidate type III secretion system effector (T3SE) genes. The five Korean strains were similar in their effectors, but differed from the 8ra strain. Across the six strains, transcription activator-like effectors (TALEs) showed diverse repeat sizes and at least six forms of the repeat variable di-residue (RVD) sequences, with differences not correlated with the origin of the strains. However, a phylogenetic tree based on the alignment of RVD sequences showed two distinct clusters with 17.5 repeats, suggesting that two distinct 17.5 RVD clusters have evolved, potentially to adapt *Xcg* to growth on distinct soybean cultivars. The predicted effector binding elements of the TALEs fell into six groups and were strongly overlapping in sequence, suggesting evolving target specificity of the binding domains in soybean cultivars. Our findings reveal the variability and adaptability of T3SEs in the *Xcg* strains and enhance our understanding of *Xcg* pathogenicity in soybean.

## 1. Introduction

*Xanthomonas citri* pv. *glycines* (*Xcg*), previously classified as *X. axonopodis* pv. *glycine* [1], causes bacterial pustules in soybean and is one of the most prevalent soybean (*Glycine max*) pathogens in the Republic of Korea [2]. *Xcg* invades and multiplies extracellularly within the apoplast, causing localized leaf spots or leaf streaks [3]. *Xcg* employs a suite of virulence factors to colonize plant tissue, including cell wall-degrading enzymes, extracellular polysaccharide and protein secretion systems [4] and a type III protein secretion system.

In the Republic of Korea, a report estimated that 89.7% of soybean fields were infected by *Xcg* in 2010 [2]. The recessive disease resistance locus *rxp* (*resistance to xanthomonas phaseoli*) confers partial resistance to *Xcg* [5] and has been introduced into many commercial soybean cultivars. However, the *Xcg* avirulence gene that triggers *rxp*-mediated resistance is unknown.

*Xcg* strains fall into distinct races [6,7,8] and exhibit varying degrees of virulence [9], which are thought to be associated with distinct profiles of transcription activator-like effectors (TALEs, [8]). The type III secretion system-dependent TALE family is unique to the *Xanthomonas* genus, although some are found in the *Ralstonia solanacearum* species complex [10]. The DNA binding domain of TALEs consists of consecutive repeats of a highly conserved 33–35-amino acid sequence. The number of repeats present in TALEs varies with an average of 17 and up to 30 repeats [3]. The amino acids at positions 12 and 13 in the DNA binding domain of TALEs are called the repeat variable di-residues (RVDs), and the sequence of RVDs determines the target specificity of TALEs [11,12]). TALEs primarily activate the transcription of plant genes involved in disease resistance or susceptibility by binding to effector-binding elements (EBEs) in their promoters [3].

Among the *Xanthomonas* TALEs identified so far, the AvrBs3 family has been extensively studied. At least one *avrBs3* gene was identified on a plasmid that is widely present in *Xcg* strains [13]. The *avrBs3* gene family is associated with mobile elements and transposase genes on the plasmids, suggesting that the *avrBs3* genes might be able to relocate within the bacterial genome. Many *Xanthomonas* strains carry *avrBs3* T3SE genes, yet the copy numbers vary depending on the species. For instance, many *X. campestris* strains carry only one *avrBs3* gene [14], whereas *X. oryzae* strains have 14 to 33 [14,15,16,17]. Based on hybridization studies, most *Xcg* strains contain three to eight AvrBs3 homologs in their genome [8,13].

Whole-genome sequences of several *Xcg* strains, including strains 12-2, CFBP 2526, CFBP 7119, and BCRC 12609, were previously reported [18,19,20]. However, these genome assemblies were generated using Illumina-based short-read data. Thus, sequence assembly of TALE genes was likely incomplete due to the presence of many repeat sequences. More recently, the complete genome sequences of three *Xcg* strains, 8ra, 12-2, and EB08 [21,22], were generated using PacBio long-read data. These strains exhibited strong conservation in their overall synteny and repertoire of AvrBs33 family effectors [22].

Our goal in this work was to compare the genomes of six additional *Xcg* strains that have various levels of virulence in soybean, namely 8ra (a strain from the United States) and the five Korean strains SL1017, SL1018, SL1045, SL1157, and K2. These Korean strains collectively represent *Xcg* isolates collected in the Republic of Korea. We aimed to identify and compare specific genetic traits associated with pathogenicity, namely the T3SEs and carbohydrate-active enzymes. We particularly focused on the TALE AvrBs3 because of its role in the resistance breeding program in Korea. Our comparative analysis uncovered variability in the sequence and number of AvrBs3 alleles in the Korean *Xcg* strains and in their presumed binding domains.

## 2. Materials and Methods

### 2.1. Bacterial Strains and DNA Preparation

Four *X. citri* pv. *glycines* (*Xcg*) strains (SL1017, SL1018, SL1045, SL1157) were selected from the 155 isolates collected in 1997 from farmers’ fields in South Korea and K2 strain was isolated in 2017. The five strains were chosen based on their diverse profiles of infection and aggressiveness on a collection of soybean cultivars and hybridization to an *avrBs3* gene family probe. A US strain, 8ra, was obtained from Dr. Heu of Seoul National University in the Republic of Korea. All strains collected were first tested using the non-host plant tomato to assess their induction of the hypersensitive reaction and the soybean cultivar, Pella, to test for pathogenicity. The taxonomic identification of the strains was verified using fatty acid methyl ester analysis and the Biolog Microbial Identification System according to the manufacturer’s instructions. For DNA extraction, *Xcg* strains were cultivated in tryptic soy agar medium (Difco, Franklin Lake, NJ, USA) at 28 °C for 24 h. High-quality, high-molecular-weight genomic DNA was prepared using a DNA extraction kit (iNtRON, Seongnam, Korea) according to the protocol for Gram-negative bacteria. The concentration of the extracted DNA was determined using a BioDrop spectrophotometer (BioDrop, Cambridge, UK). The integrity of DNA was checked by agarose gel electrophoresis and quantified using a Qubit 2.0 fluorometer (Invitrogen, Carlsbad, CA, USA).

### 2.2. Genome Sequencing and Assembly

DNA samples were sequenced using PacBio single-molecule real-time (SMRT) technology. A total of 5 μg for each sample was used as input for library preparation. Libraries were constructed with the SMRTbell Template Prep Kit 1.0 (PN 100-259-100) following the manufacturer’s instructions (Pacific Biosciences, Menlo Park, CA, USA). Fragments of the SMRTbell template that were less than 20 kb were removed using the Blue Pippin Size selection system. The constructed library was validated to be a large-insert library using an Agilent 2100 Bioanalyzer. After a sequencing primer was annealed to the SMRTbell template DNA, DNA polymerase was bound to the complex using the DNA Polymerase Binding kit P6. This polymerase-SMRTbell-adaptor complex was then loaded onto the SMRT cells. The SMRTbell library was sequenced using one SMRT cell with the MagBead OneCellPerWell v1 Protocol (insert size 20 kb, movie time 1 × 240 min) using P6-C4 chemistry (DNA sequencing Reagent 4.0) on the PacBio RS II (Pacific Biosciences) sequencing platform.

De novo genome assembly was conducted using the hierarchical assembly pipeline of HGAP2 (ver.2.3.0) software implemented in PacBio SMRTLink. PRODIGAL version 2.6.2 [23] was used to predict protein coding sequences (CDSs) based on the bacterial genome database. Rfam and tRNAscan-SE were used to identify rRNA and tRNA sequences, respectively, in the assembled genomes. Each of the *Xanthomonas* genome sequences in this study was assigned an NCBI GenBank accession number as listed in Table 1.

### 2.3. Comparative Genome Analysis

The genome sequences of the six strains were generated and assembled for comparative analysis. To identify homology patterns among the *Xcg* genomes, multiple genome sequences were aligned with progressive Mauve algorithm implemented in the Mauve software3 [24]. Genome and pan-genome orthologous groups (POGs) of genes were identified by clustering genomes with the unweighted pair group method with arithmetic mean (UPGMA) method, and a heat map was generated to reflect the presence and absence of genes.

For analysis of POGs, a pairwise gene-to-gene comparison of each genome was conducted using USEARCH [25], and the genes were compared using a reciprocal homology search. A pairwise orthologous group was defined after the initial grouping, and partial genes that were left out of groups due to their short sequence length were targeted for clustering analysis against the POGs using UCLUST (≥95% identity). The CDSs were classified into groups based on their predicted functions, with reference to orthologous groups (EggNOG 4.5, [26]). The annotation of each CDS was conducted by a homology search against the Swiss-Prot, EggNOG 4.1, SEED (the database and infrastructure for comparative genomics), and Kyoto Encyclopedia of Genes and Genomes (KEGG) databases.

### 2.4. CAZyme and TALE Analysis

Carbohydrate active enzymes (CAZymes) were evaluated with the online version of automated CAZyme annotation using the dbCAN meta server (http://bcb.unl.edu/dbCAN2/, accessed date: 23 June 2021), which implements three independent search modules, i.e., HMMER, Diamond, and Hotpop. All predicted genes identified by the Patric 3.6 annotation pipeline [27] were used to search and identify effectors by running a local BLAST using the Blast2go version 3.2. These predicted genes were further used to identify TALE genes using the HMMER3 program to search the query sequences against the Pfam 27.0 database, in which separate hidden Markov models were constructed using HMMER based on their amino acid sequences. AnnoTALE [28] was used for the analysis and annotation of TALE genes and for clustering similar TALEs into classes with agglomerative hierarchical clustering using average linkage. For automated extraction of RVDs, the corresponding protein sequence was aligned to the consensus repeat, and the RVDs comprised of the 12th and 13th amino acid positions of each repeat were extracted. For this alignment, a BLOSUM62 substitution matrix and affine gap costs with gap open penalty of 12 and gap extension penalty of 3 were used to account for the existence of aberrant repeat lengths of greater than 35 amino acids. RVDs were aligned using Geneious alignment (Geneious version 11.1.5). The relationship tree based on RVDs was constructed using a Neighbor-Joining method implemented in Geneious Prime (version 11.1.5). Bootstrapping of 1000 replicates was applied to check out the robustness of each clade. Putative target sites of a given TALE were determined by the binding specificity of the RVD using a TALE target site finder web application, TALgetter v2.3, in a local Galaxy server [29] (https://www.jstacs.de/index.php/TALgetter, accessed date: 23 June 2021).

## 3. Results

### 3.1. Whole-Genome Sequence Analysis of Korean X. citri pv. glycines Strains

To gain insights into the genome diversity of *Xcg* strains we conducted genome sequence analysis of five Korean *Xcg* strains (K2, SL1017, SL1018, SL1157, and SL1045) and the 8ra strain from the US [22]. Long-read sequence data obtained from the PacBio sequencing platform were used to characterize and compare the genomes of the six strains. De novo assembly of the *Xcg* sequences resulted in two to five contigs per strain with an average coverage of 157x. The assembled genome sizes ranged from 5.2 (SL1018) to 5.4 Mbp (8ra) with GC contents ranging from 64.6 to 64.7% (Table 1). The number of predicted total CDSs varied from 4314 in SL1018 to 4597 in 8ra (Table 1).

**Figure 1:**
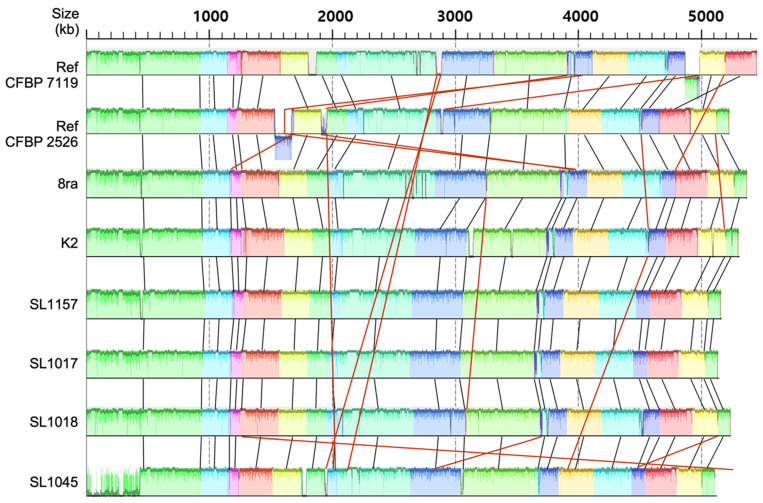
Whole-genome comparison of eight *X. citri* pv. *glycines* strains by Progressive Mauve alignment. CFBP 7119 and CFBP 2526, isolated in Sudan and Brazil, respectively, were used as reference genomes for comparison [19]. The scale represents coordinates of each genome. Different color blocks represent LCBs (Local Collinear Blocks), which are conserved segments within the genomes. Within a LCB, the white area represents low similarity regions or regions unique to one genome but absent in another. Colored lines indicate rearrangement of LCBs between the genomes.

To examine the conservation and variation in the *Xcg* genomes, we performed Progressive Mauve alignment (Figure 1), which shows the conserved genomic clusters as similarly colored blocks among the six fully sequenced *Xcg* genomes. The genomes of the sequenced *Xcg* strains in this study (K2, SL1017, SL1018, SL1045, SL1157, and 8ra) are highly homologous to the reference genomes CFBP 7119 and CFBP2526, although small genomic rearrangements and inversions were identified (Figure 1). Two other strains, 12-2 and EB08, whose genome sequences are available, were not included because they are nearly identical to strain 8ra (Carpenter et al., 2019) [22]. The Progressive Mauve alignment demonstrated that the genomes of 8ra, K2, SL1017, SL1018, SL1045, and SL1157 were similar and closely related (Figure 1). These strains were co-linear along the chromosomes; however, SL1045 showed a different genomic arrangement than the other strains.

### 3.2. Comparison of Gene Content and Number of Korean X. citri pv. glycines Strains

**Figure 2:**
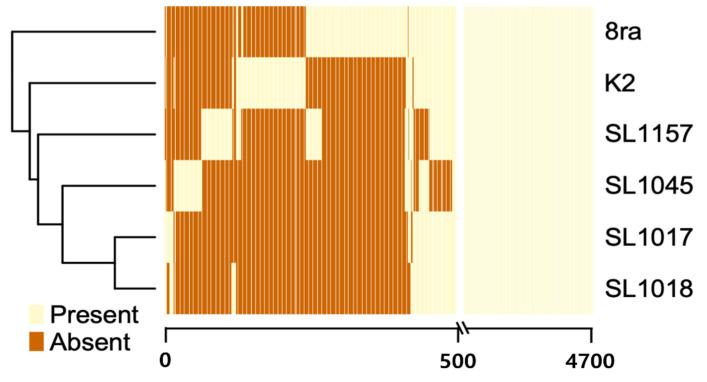
Comparison of gene content (presence/absence) as shown in a heatmap and a dendogram (unweighted pair group method with arithmetic mean (UPGMA)) of 4,700 protein-coding sequences (CDSs) that were placed into pan-genome orthologous groups (POGs). The numbers on the X-axis and columns in the figure represent distinct POGs, which were ordered based on their differential presence (indicated in yellow) or absence (indicated in orange) among the strains.

We constructed a heatmap and dendrogram based on the gene content of the six *Xcg* strains. The 4700 CDSs in the strains were clustered into pan-genome orthologous groups (POGs) (Figure 2). The heatmap showed that the presence/absence patterns of the POGs in the strain 8ra were different from those in the Korean strains since there were about 100 extra genes in 8ra. Compared to strain 8ra, SL1017 and SL1018 showed 0.38% variation, while K2 showed 2% variation. SL1045 showed 4% variation. In the UPGMA relationship tree based on gene sharing between strains, SL1017 was closely aligned with SL1018 and 8ra was closely aligned with K2.

**Figure 3:**
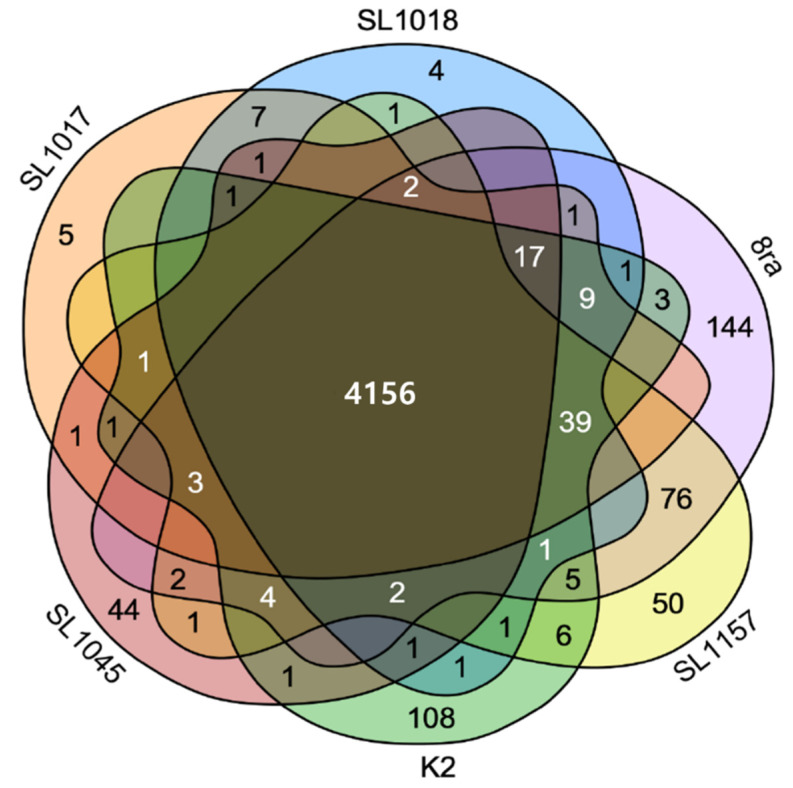
Venn diagram of genes found within the five Korean strains and a US strain, 8ra, with numbers in the intersected regions indicating shared genes.

We also constructed a Venn diagram [30] to compare predicted gene numbers in the five *Xcg* strains and 8ra (Figure 3) and identify the number of shared and unique CDSs. The six *Xcg* strains were closely related to each other at the genome sequence level; they shared 4156 genes (CDSs), with an average of 260 shared genes per genome. SL1017 and SL1018 appeared to have five and four unique genes, respectively. SL1157, SL1045, and K2 had 50, 44, and 108 unique genes, respectively. Strain 8ra had 144 unique genes (Figure 3). Excluding SL1045, the strains shared 39 genes, with 8ra and SL1157 sharing the most, 76 genes.

### 3.3. Functional Gene Distribution in the Korean X. citri pv. glycines Strains and Strain 8ra

**Figure 4:**
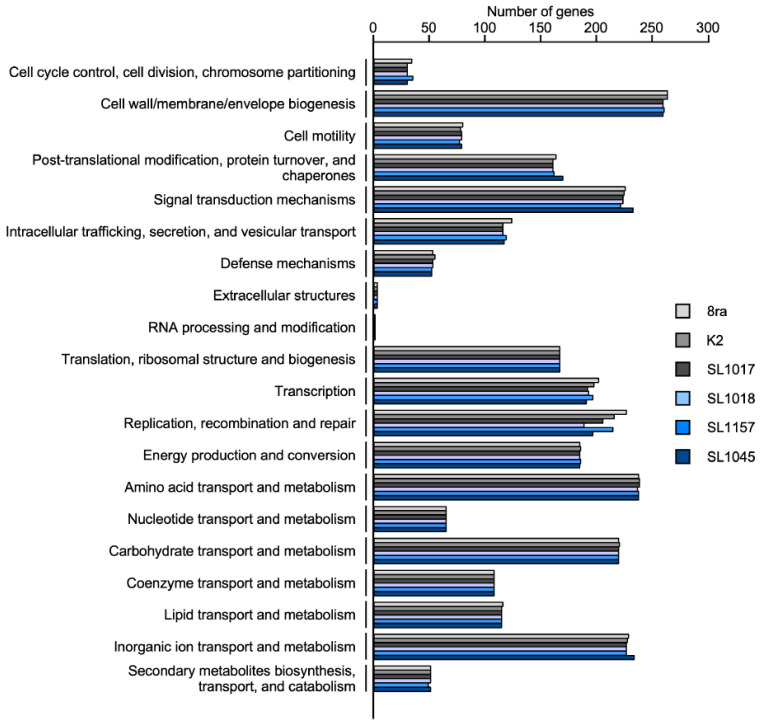
Number of genes in distinct functional categories for the five Korean *Xcg* strains and the US *Xcg* strain 8ra. All putative genes were aligned to the database of Clusters of Orthologous Groups (COGs) to predict possible functions. The genes were classified into 21 total COGs. The unknown function category is not shown. Values on the X-axis indicate the number of genes, and the Y-axis labels indicate the COG categories.

To compare the distribution of functional genes among the six *Xcg* strains in relation to bacterial virulence, we analyzed the functional gene categories in the Clusters of Orthologous Groups of proteins (COGs) database [31] (Figure 4). Among the predicted CDSs, about 70% of the genes were classified into one of the 21 COGs categories, and about 30% were of unknown function. In five functional categories, COGs were similarly distributed; these groups were the extracellular structures, RNA processing and modification group, translation, ribosomal structure and biogenesis group, nucleotide transport and metabolism, and coenzyme transport and metabolism group. The strains showed variability in gene number, primarily in two categories, transcription and replication, recombination, and repair. Strain SL1045 had more genes in several categories such as post-translational modifications, protein turnover, and chaperones, signal transduction mechanisms, and inorganic ion transport and metabolism.

### 3.4. Comparison of Total Number of CAZymes Present in the Genomes of Korean X. citri pv. glycines Strains and Strain 8ra

**Figure 5:**
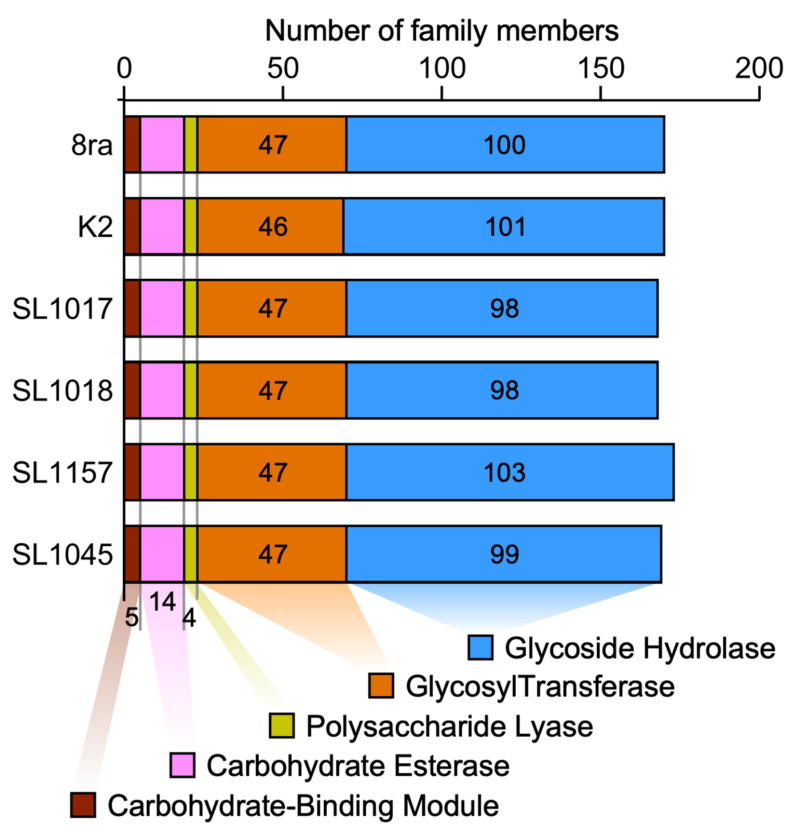
Comparison of total number of CAZymes predicted in the genomes of six *X. citri* pv. *glycines* strains. CAZymes were classified into five classes of enzymatic domains, namely, the glycosyl hydrolase (GH) domain class, glycosyl transferase (GT) domain class, polysaccharide lyase (PL) domain class, carbohydrate esterase (CE) domain class and carbohydrate binding module (CBM) domain class.

To examine the repertoire of carbohydrate-active enzymes in the *Xcg* strains, we collected CAZymes from five classes and organized them into families based on their amino acid sequence similarity. We found that the strains had similar proportions of CAZymes belonging to the five major functional classes (Figure 5). Among the CAZymes identified, 58–59% were glycoside hydrolases (GHs), 27.5% were glycosyltransferases (GTs), 2% were polysaccharide lyases (PLs), 8% were carbohydrate esterases (CEs), and 2% were carbohydrate-binding modules (CBMs). All of the strains had four PLs, 14 CEs, and five CBMs, whereas they differed primarily in the number of GHs. SL1157 had the highest number of GHs.

### 3.5. Comparative Analysis of Type III Secretion System Effector Candidate Genes

**Figure 6:**
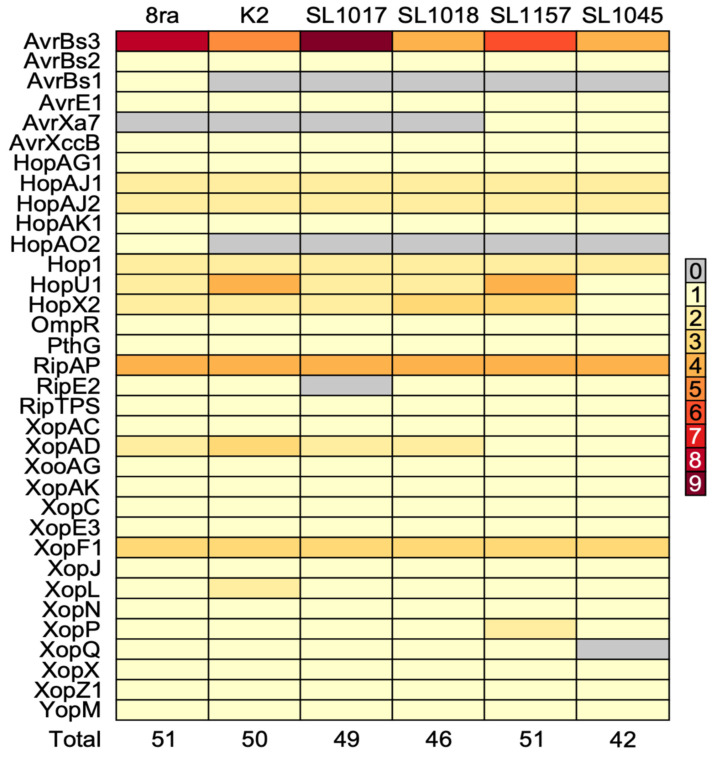
Number of type III secretion system effectors in the five Korean *X. citri* pv. *glycines* strains and the US strain 8ra. The colors represent the number of genes for each effector.

The type III secretion system (T3SS) is critical to the pathogenicity of *Xcg*, as it secretes effectors into the host plant cell. To identify T3SS effectors (T3SEs), we used the predicted genes for each of the six strains from Patric 3.6 as queries for BLAST searches of *Xcg* using the Blast2go program. The identified effector names and functions are summarized in Figure 6. The comparative analysis of T3SE candidate genes showed the differences among the strains. A total of 49 effectors were identified in 8ra, 44 effectors in K2, 47 effectors in SL1017, 40 effectors in SL1045, 49 effectors in SL1157, and 44 effectors in SL1018. The US strain 8ra had two effectors, AvrBs1 and HopAO2, that were not found in the five Korean strains. Park et al. (2008) [8] also showed that strains K2, SL1017, SL1018, SL1045, and SL1157 lack AvrBs1. The strains share few *Xop* effectors with strains causing bacterial spot of tomato and pepper (*X. euvesicatoria* and *X. perforans* strains); this includes both host-specific and non-host-specific effectors [32]. Additionally, SL1017 and SL1045 did not have RipE2 and XopQ, respectively, and SL1157 and SL1045 had AvrXa7.

### 3.6. Comparative Analysis of Transcription Activator-like Effector Candidate Genes

Transcription activator-like effectors (TALEs) are critical for virulence in *Xanthomonas* spp., with some activating key susceptibility genes. Here, we extracted the sequences of TALEs, all of which are homologs of AvrBs3 (Figure 6), to examine the differences among the *Xcg* strains. As TALE specificity primarily depends on the repeat variable di-residues, or RVDs, we aligned the RVD sequences and analyzed the TALEs of each strain with both the HMMER3 program using the Pfam 27.0 database and the AnnoTALE program (Supplementary Table S1). The HMMER3 program detected more TALEs than the AnnoTALE program [28]. To compare TALEs in the five Korean *Xcg* strains and 8ra, we renamed all complete TALEs with the guidance of AnnoTALE (Table 2).

**Figure 7:**
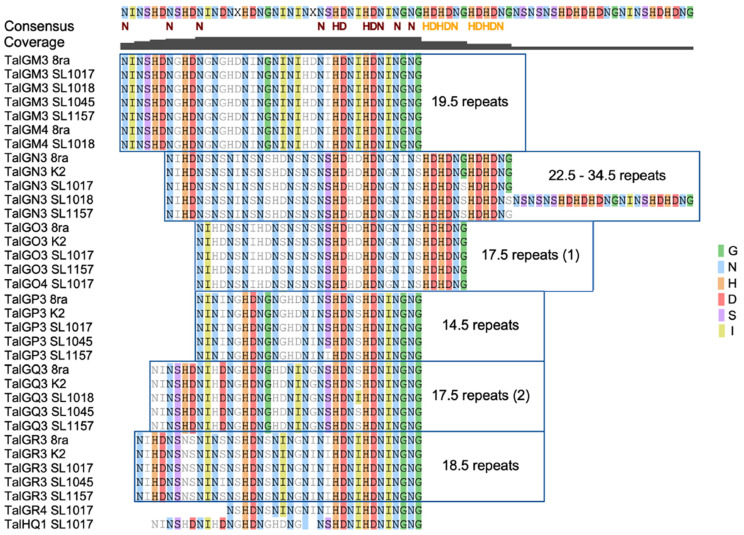
Protein sequence alignment for the repeat variable di-residues (RVDs) from the transcription activator-like effectors (TALEs) of six *X. citri* pv. *glycines* strains. All RVDs of TALEs were aligned with Geneious Alignment (https://www.geneious.com, accessed date: 23 June 2021) (Supplementary Table S2); only those predicted to be complete are shown here. Red lines above the RVD sequence highlight recurring repeats of the sequence HDN.

**Figure 8:**
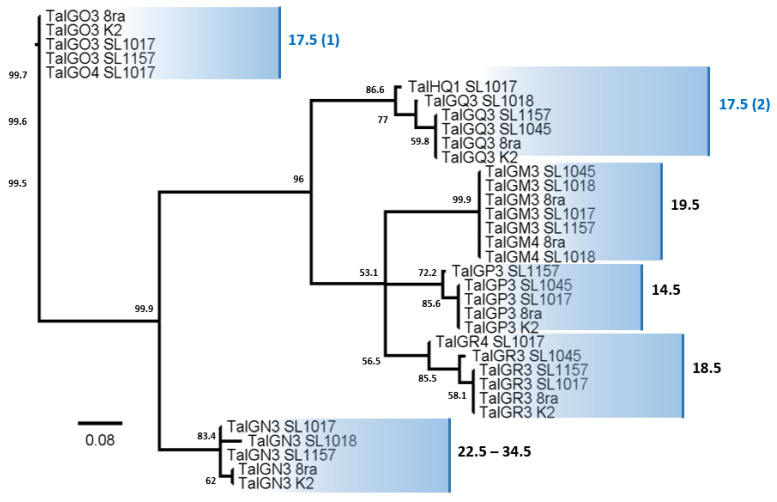
Phylogenetic tree of the transcription activator-like effectors identified in six *X. citri* pv. *glycines* strains based on their repeat variable di-residue sequences. The Neighbor-Joining tree was based on bootstrapping of 1000 replications. Bootstrap values above 50% are shown on the node. Bar (0.08) indicates the substitution rate per nucleotide position.

Given that some of the TALEs appear to have incomplete forms of RVDs (Supplementary Table S1), we used only TALE candidates with over 12.5 repeats for RVD analysis. Among these TALEs that were considered complete, the number of repeats varied from 12.5 to 34.5 (Table 2). These TALEs were classified into six groups (Figure 7). Alignment of the RVDs of all of the TALEs showed the presence of a 21-residue consensus region, within which N and HDN residues were conserved in all complete TALEs (Figure 8). The TALE proteins of the *Xcg* strains clustered based on the repeat number, with the exception of the TALEs containing 17.5 repeats, which formed two groups (Figure 7). The most common TALEs had 17.5 repeats, followed by those with 19.5 repeats. The sequence HDN recurred frequently within the TALEs, with each TALE group containing at least four HDN repeats (Figure 8).

A phylogenetic tree constructed based on the RVDs of the TALEs showed clustering into three major groups, one with TALEs containing 17.5 repeats (group 1), one with TALEs containing 22.5–34.5 repeats, and one with all of the other TALEs. The phylogenetic tree further supports the division of the TALEs with 17.5 repeats into two distinct clades. 

### 3.7. Comparative Analysis of the Predicted Effector Binding Elements of the Transcription Activator-like Effector Candidate Genes

**Figure 9:**
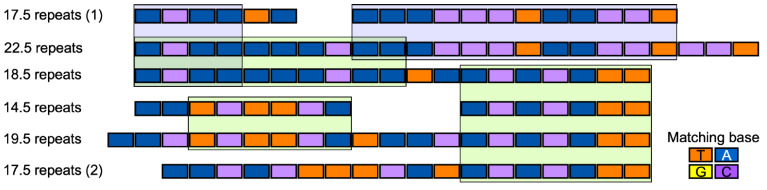
The predicted effector binding elements (EBEs) of the transcription activator-like effectors (TALEs) of six *X. citri* pv. *glycines* strains. The EBEs were predicted based on the binding specificity of the repeat variable di-residue sequences with a web-application of TALE target site finder, TALgetter v2.3 in a local Galaxy server (https://www.jstacs.de/index.php/TALgetter, accessed date: 23 June 2021). Five conserved domains are highlighted with shaded boxes.

Since the prediction of the effector binding elements (EBEs) is essential to elucidating the biological functions of TALEs, we predicted the EBEs of the TALEs of the six *Xcg* strains. TALE target sites were predicted using TALgetter [29]. The target gene promoter sequences contained exclusively A, T, and C bases (Figure 9). The predicted EBEs showed five conserved domains (Figure 9). These domains were intermixed among the TALEs. For example, EBEs corresponding to TALEs with 18.5 repeats shared one domain with those containing 22.5 repeats and one domain with those containing 14.5, 19.5, and 17.5 repeats (second group).

## 4. Discussion

*Xcg* is an emerging problematic pathogen in soybean in Korea. Genome-wide studies of pathogenicity effectors and analysis of the pathogenic variability of representative strains in Korea should allow us to understand the extent of variation among distinct races of *Xcg*. Here, we characterized potential pathogenicity factors in five Korean *Xcg* strains which were previously reported to exhibit distinct infection profiles and aggressiveness on soybean. Specifically, we analyzed whole genomes sequences of these five Korean strains and the US strain 8ra for two types of pathogenicity factors, plant cell wall-degrading enzymes and type III secretion system effectors.

In a Progressive Mauve alignment, the genomes of the six strains, 8ra, K2, SL1017, SL1018, SL1045, and SL1157, were similar and closely related to the genomes of two reference strains, CFBP 7119 and CFBP2526, although the SL1045 genome was >100 kb smaller than the others. Up to 100 genes are missing in strain SL1045 compared with the other strains, accounting for its heterogeneous gene content (Figure 2 and Figure 4).

Many members of the *Xanthomonas* species are mesophyll pathogens, and their genomes generally encode a range of plant cell wall-degrading enzymes and the associated type II secretion systems encoded by the *xps* and *xcs* gene clusters. Genes for such carbohydrate-degrading enzymes, or CAZymes, have been identified previously in strains of bacterial leaf spot-causing *Xanthomonas* species [33]. Here, we found that the *Xcg* strains examined had a similar number of CAZymes in each of the distinct domain classes, with some variation in the number of GHs. The GHs in these *Xcg* strains include GHs in the GH9 and GH5 families. This finding is consistent with the reported role for xyloglucan in the transcriptional activation of virulence factors in *Xanthomonas* spp. [34], since xyloglucan is used by GH9 and GH5 enzymes with xyloglucanase activity. Notably, the sugars released by these enzymes elicit the expression of several key virulence factors in *Xanthomonas* spp., including components of the type III secretion system.

A previous study reported that the Korean *Xcg* strains do not have AvrBs1 homologs whereas strain 8ra does [8]. Here, we found that the Korean *Xcg* strains also do not have HopAO2 homologs whereas strain 8ra does. HopAO2 effectors have phosphatase activity and are involved in the suppression of plant immune responses [35]. The AvrBs1 and HopAO2 effectors may facilitate survival of strain 8ra inside plant cells and thus contribute to its pathogenicity. To date, only a few Korean isolates have been analyzed, so continuous screening of recently isolated *Xcg* strains from the Republic of Korea will help indicate if strains with either of these two effectors are currently present or, in the future, emerge or are introduced into the country.

Distinct host specificity has been reported among strains that carry different numbers of TALE homologs and that differ in their response to diverse soybean cultivars [8]. Athinuwat et al. (2009) [6] also showed that differences in the virulence of *Xcg* strains to soybean cultivars is determined by AvrBs3. Since the TALE AvrBs3 determines host and virulence specificity, identifying the target specificity of AvrBs3 variants with distinct sequences and repeats could provide insights into AvrBs3 functions. In this study, a collection of AvrBs3 homologs revealed that these TALEs contain repeat domains of various lengths and fall into six groups based on their RVD sequences. Schandry et al. [36] showed that TALEs in *Ralstonia solanacearum* could be similarly grouped into subclasses based on RVD sequences. Our study showed that the RVDs in these AvrBs3 homologs were nearly perfectly conserved within a group. Moreover, N and HDN residues make up a conserved core in all of the examined *Xcg* TALEs. The presence of HDN in the core of *Xcg* TALEs is similar to the finding that HDN residues form a conserved core in almost all Avr/Pth proteins in *X. oryzae* pv. *oryzae*, including in AvrBs3 homologs [37]. The *Xcg* TALE classes with 22.5 repeats and 33.5 repeats had two and four HDHDN repeats, respectively, which suggests that they may have resulted from a duplication event. An analysis of 113 TALEs showed that TALEs with 17.5 repeats were the most common, followed by those with 19.5 and 15.5 repeats [3]. Our results with the *Xcg* strains were similar in that TALEs with 17.5 repeats were the most common, followed by those with 19.5 and 18.5 repeats; all of the *Xcg* strains had at least one TALE with 18.5 repeats. Also, we found that the phylogenetic tree of TALEs based on RVDs divided the TALEs with 17.5 repeats into two clades, suggesting that two distinct 17.5-repeat-containing TALEs have evolved; these may exhibit specificity to distinct soybean cultivars.

Genes for the *Xcg* TALEs with 19.5 repeats were present on plasmids in the *Xcg* strains (Supplementary Table S3). Since 85% of isolates from Korea contained genes necessary for plasmid transfer and mobilization [13], a plasmid with a TALE gene could potentially transfer it to other strains, with subsequent integration of the TALE gene into the chromosome. Gochez et al. (2018) [38] emphasized that since TALE genes are located on plasmids in *Xcg* strains, the variability of TALEs and the shuffling of plasmids among the pathogen population may contribute to pathogen variability and adaptive evolution of the host. An analysis of the variability and distribution of TALEs on plasmids in more strains of *Xcg* may provide a better understanding of the evolution of TALEs in this pathogen.

Following the secretion of TALEs into the cells of a plant host, TALEs may enter the host cell nucleus and activate specific genes by binding to effector binding elements (EBEs) in the promoter [39]. Accurate prediction of EBEs is essential for the robust design of artificial TALE DNA-binding domains in biotechnological applications, and for gaining insights into the host cell TALE targets. In this study, we used the sequence of the RVDs to predict the EBEs of the *Xcg* TALEs. We found that, surprisingly, the predicted EBEs of the *Xcg* TALEs contained only A, T and C bases, but not G; this contrasts with the EBEs in other genera such as *Xanthomonas oryzae* pv. *oryzae*, *Xanthomonas oryzae* pv. *oryzicola* [3], and *Ralstonia solanacearum* [35]. Another study found that EBE diversity from four *Ralstonia solanacearum* pathotypes was limited [35]. We also found that the EBEs of the *Xcg* TALEs formed six distinct groups that had domains that were intermixed and targeted by multiple TALEs, suggestive of recombination events occurring during the evolution of these AvrBs3 homologs. 

In summary, we generated whole-genome sequence assemblies of six *Xcg* strains that exhibit various levels of virulence in soybean. We did not find significant differences among these strains in the genes falling into distinct functional classes or in their carbohydrate-degrading enzymes. We found that one strain, the US strain 8ra, had two more T3SEs than the five Korean strains examined, and that overall, the greatest variation in their T3SEs was in the TALE effectors, namely in the AvrBs3 homologs. In looking at the variability in the structure and predicted binding domains of these AvrBs3 homologs, we found multiple classes that vary in the number of repeat regions; furthermore, variation in the predicted binding domains suggests recombination, domain swapping, and likely potential adaptation of these TALEs to their hosts. This comparative genome analysis thus highlights a high overall similarity among *Xcg* strains and a high level of variation in TALE effectors suggestive of duplication and subsequent adaptation to distinct soybean cultivars.

## Figures and Tables

**Table 1:** General genomic features of six *X. citri* pv. *glycines* strains.

Strain	No. of Contigs	Depth of Coverage (x)	Genome Size (bp)	% GC	No. of CDSs	No. of Unique CDSs	NCBI Accession Number
8ra	2	178	5,426,838	64.6	4597	144	CP041781, CP041782
K2	2	108	5,322,598	64.6	4519	108	CP041966, CP041967
SL1017	3	198	5,197,177	64.7	4341	5	VMHQ00000000
SL1018	2	163	5,162,305	64.7	4314	5	CP041961, CP041962
SL1157	2	175	5,292,354	64.6	4490	4	CP041963, CP041964
SL1045	5	123	5,169,163	64.7	4332	44	VMHR00000000

**Table 2:** The transcription activator-like effectors (TALEs) that were identified in six *Xcg* strains and annotated using AnnoTALE based on the repeat variable di-residues (RVDs).

Strain Name	AnnoTALE Name	No. of Repeats	RVDs (the 12th and 13th Amino Acid Residues of Each Repeat Region)
8ra	TalGN3 8ra	22.5	NI-HD-NS-NS-NI-NS-NS-HD-NS-NS-NS-HD-HD-HD-NG-NI-NS-HD-HD-NG-HD-HD-NG
	TalGR3 8ra	18.5	NI-HD-NS-NS-NI-NS-NS-HD-NS-NI-NG-NI-NI-HD-NI-HD-NI-NG-NG
	TalGO3 8ra	17.5	NI-HD-NS-NI-HD-NS-NS-NS-NS-HD-HD-HD-NG-NI-NS-HD-HD-NG
	TalGQ3 8ra	17.5	NI-NS-HD-NI-HD-NG-HD-NG-HD-NI-NG-NS-HD-NS-HD-NI-NG-NG
	TalGP3 8ra	14.5	NI-NI-NG-HD-NG-NG-HD-NI-NS-HD-NS-HD-NI-NG-NG
	TalGM3 8ra	19.5	NI-NS-HD-NG-HD-NG-NG-HD-NI-NG-NI-NI-HD-NI-HD-NI-HD-NI-NG-NG
	TalGM4 8ra	19.5	NI-NS-HD-NG-HD-NG-NG-HD-NI-NG-NI-NI-HD-NI-HD-NI-HD-NI-NG-NG
K2	TalGN3 K2	22.5	NI-HD-NS-NS-NI-NS-NS-HD-NS-NS-NS-HD-HD-HD-NG-NI-NS-HD-HD-NG-HD-HD-NG
	TalGR3 K2	18.5	NI-HD-NS-NS-NI-NS-NS-HD-NS-NI-NG-NI-NI-HD-NI-HD-NI-NG-NG
	TalGO3 K2	17.5	NI-HD-NS-NI-HD-NS-NS-NS-NS-HD-HD-HD-NG-NI-NS-HD-HD-NG
	TalGQ3 K2	17.5	NI-NS-HD-NI-HD-NG-HD-NG-HD-NI-NG-NS-HD-NS-HD-NI-NG-NG
	TalGP3 K2	14.5	NI-NI-NG-HD-NG-NG-HD-NI-NS-HD-NS-HD-NI-NG-NG
SL1017	TalGN3 SL1017	22.5	NI-HD-NS-NS-NI-NS-NS-HD-NS-NS-NS-HD-HD-HD-NG-NI-NS-HD-HD-NS-HD-HD-NG
	TalGO3 SL1017	17.5	NI-HD-NS-NI-HD-NS-NS-NS-NS-HD-HD-HD-NG-NI-NS-HD-HD-NG
	TalHQ1 SL1017	16.5	NI-NS-HD-NI-HD-NG-HD-NG-HD-NG-NS-HD-NI-HD-NI-NG-NG
	TalGP3 SL1017	14.5	NI-NI-NG-HD-NG-NG-HD-NI-NS-HD-NS-HD-NI-NG-NG
	TalGR4 SL1017	12.5	NS-HD-NS-NI-NG-NI-NI-HD-NI-HD-NI-NG-NG
	TalGM3 SL1017	19.5	NI-NS-HD-NG-HD-NG-NG-HD-NI-NG-NI-NI-HD-NI-HD-NI-HD-NI-NG-NG
	TalGR3 SL1017	18.5	NI-HD-NS-NS-NI-NS-NS-HD-NS-NI-NG-NI-NI-HD-NI-HD-NI-NG-NG
	TalGO4 SL1017	17.5	NI-HD-NS-NI-HD-NS-NS-NS-NS-HD-HD-HD-NG-NI-NS-HD-HD-NG
SL1018	TalGN3 SL1018	34.5	NI-HD-NS-NS-NI-NS-NS-HD-NS-NS-NS-HD-HD-HD-NG-NI-NS-HD-HD-NS-HD-HD-NS-NS-NS-NS-HD-HD-HD-NG-NI-NS-HD-HD-NG
	TalGQ3 SL1018	17.5	NI-NS-HD-NI-HD-NG-HD-NG-HD-NI-NG-NS-HD-NI-HD-NI-NG-NG
	TalGM3 SL1018	19.5	NI-NS-HD-NG-HD-NG-NG-HD-NI-NG-NI-NI-HD-NI-HD-NI-HD-NI-NG-NG
	TalGM4 SL1018	19.5	NI-NS-HD-NG-HD-NG-NG-HD-NI-NG-NI-NI-HD-NI-HD-NI-HD-NI-NG-NG
SL1157	TalGN3 SL1157	22.5	NI-HD-NS-NS-NI-NS-NS-HD-NS-NS-NS-HD-HD-HD-NG-NI-NS-HD-HD-NS-HD-HD-NG
	TalGO3 SL1157	17.5	NI-HD-NS-NI-HD-NS-NS-NS-NS-HD-HD-HD-NG-NI-NS-HD-HD-NG
	TalGQ3 SL1157	17.5	NI-NS-HD-NI-HD-NG-HD-NG-HD-NI-NG-NS-HD-NS-HD-NI-NG-NG
	TalGP3 SL1157	14.5	NI-NI-NG-HD-NG-NG-HD-NI-NI-HD-NS-HD-NI-NG-NG
	TalGM3 SL1157	19.5	NI-NS-HD-NG-HD-NG-NG-HD-NI-NG-NI-NI-HD-NI-HD-NI-HD-NI-NG-NG
	TalGR3 SL1157	18.5	NI-HD-NS-NS-NI-NS-NS-HD-NS-NI-NG-NI-NI-HD-NI-HD-NI-NG-NG
SL1045	TalGM3 SL1045	19.5	NI-NS-HD-NG-HD-NG-NG-HD-NI-NG-NI-NI-HD-NI-HD-NI-HD-NI-NG-NG
	TalGR3 SL1045	18.5	NI-HD-NS-NS-NI-NI-NS-HD-NS-NI-NG-NI-NI-HD-NI-HD-NI-NG-NG
	TalGQ3 SL1045	17.5	NI-NS-HD-NI-HD-NG-HD-NG-HD-NI-NG-NS-HD-NS-HD-NI-NG-NG
	TalGP3 SL1045	14.5	NI-NI-NG-HD-NG-NG-HD-NI-NS-HD-NS-HD-NI-NG-NG

## Data Availability

Data sharing is not applicable to this article.

## Supplementary Materials

The following are available online at https://www.mdpi.com/article/10.3390/microorganisms9102065/s1, Table S1: The repeat variable di-residues (RVDs) from all transcription activator-like effectors of six *Xanthomonas campestris* pv. *glycines* strains using both the HMMER3 program with the Pfam 27.0 database and AnnoTALE program, Table S2: Clustering of the RVDs from all of the TALEs in Table S1 into six groups based on sequence similarity using the Geneious Alignment program, Table S3: RVDs of *X. campestris* pv. *glycines* TALEs found on plasmids.
