# Supplementary material for: Pan-Genome Analysis of Effectors in Korean Strains of the Soybean Pathogen Xanthomonas citri pv. glycines
Source: Microorganisms. 2021 Sep 30;9(10):2065. doi: 10.3390/microorganisms9102065 (PMC8538872; doi:10.3390/microorganisms9102065)
Supplement: Supplementary file 1 [file microorganisms-09-02065-s001.zip › microorganisms-1372358-supplementary.pdf]

Supplementary Table S1. The repeat variable di-residues (RVDs) from all transcription activator-like effectors (TALEs) of six *Xanthomonas citri* pv. *glycines* strains using both the HMMER3 program with the Pfam 27.0 database and AnnoTALE program[1].

strain name	AnnoTALE name	HMMER3	No. of Repeats	RVDs (the 12th and 13th amino acid residues of each repeat region)
8ra	TalGN3 8ra	8ra_1	22.5	NI-HD-NS-NS-NI-NS-NS-HD-NS-NS-NS-HD-HD-HD-NG-NI-NS-HD-HD-NG-HD-HD-NG
	TalGM3 8ra	8ra_2	19.5	NI-NS-HD-NG-HD-NG-NG-HD-NI-NG-NI-NI-HD-NI-HD-NI-HD-NI-NG-NG
	TalGM4 8ra	8ra_3	19.5	NI-NS-HD-NG-HD-NG-NG-HD-NI-NG-NI-NI-HD-NI-HD-NI-HD-NI-NG-NG
	TalGR3 8ra	8ra_4	18.5	NI-HD-NS-NS-NI-NS-NS-HD-NS-NI-NG-NI-NI-HD-NI-HD-NI-NG-NG
	TalGO3 8ra	8ra_5	17.5	NI-HD-NS-NI-HD-NS-NS-NS-NS-HD-HD-HD-NG-NI-NS-HD-HD-NG
	TalGQ3 8ra	8ra_6	17.5	NI-NS-HD-NI-HD-NG-HD-NG-HD-NI-NG-NS-HD-NS-HD-NI-NG-NG
	TalGP3 8ra	8ra_7	14.5	NI-NI-NG-HD-NG-NG-HD-NI-NS-HD-NS-HD-NI-NG-NG
	TalED3 8ra	8ra_8	4.5	NI-HD-NI-NG-NG
K2	TalGN3 K2	K2_1	22.5	NI-HD-NS-NS-NI-NS-NS-HD-NS-NS-NS-HD-HD-HD-NG-NI-NS-HD-HD-NG-HD-HD-NG
	TalGR3 K2	K2_2	18.5	NI-HD-NS-NS-NI-NS-NS-HD-NS-NI-NG-NI-NI-HD-NI-HD-NI-NG-NG
	TalGO3 K2	K2_3	17.5	NI-HD-NS-NI-HD-NS-NS-NS-NS-HD-HD-HD-NG-NI-NS-HD-HD-NG
	TalGQ3 K2	K2_4	17.5	NI-NS-HD-NI-HD-NG-HD-NG-HD-NI-NG-NS-HD-NS-HD-NI-NG-NG
	TalGP3 K2	K2_5	14.5	NI-NI-NG-HD-NG-NG-HD-NI-NS-HD-NS-HD-NI-NG-NG
		K2_6	7	NI-NI-HD-NI-HD-NI-HD
		K2_7	3.5	NI-NS-HD-NG
		K2_8		
SL1017	TalGN3 SL1017	SL1017_1	22.5	NI-HD-NS-NS-NI-NS-NS-HD-NS-NS-NS-HD-HD-HD-NG-NI-NS-HD-HD-NS-HD-HD-NG
	TalGM3 SL1017	SL1017_2	19.5	NI-NS-HD-NG-HD-NG-NG-HD-NI-NG-NI-NI-HD-NI-HD-NI-HD-NI-NG-NG
	TalGR3 SL1017	SL1017_3	18.5	NI-HD-NS-NS-NI-NS-NS-HD-NS-NI-NG-NI-NI-HD-NI-HD-NI-NG-NG
	TalGO3 SL1017	SL1017_4	17.5	NI-HD-NS-NI-HD-NS-NS-NS-NS-HD-HD-HD-NG-NI-NS-HD-HD-NG
	TalGO4 SL1017	SL1017_5	17.5	NI-HD-NS-NI-HD-NS-NS-NS-NS-HD-HD-HD-NG-NI-NS-HD-HD-NG
	TalHQ1 SL1017	SL1017_6	16.5	NI-NS-HD-NI-HD-NG-HD-NG-HD-NG-NS-HD-NI-HD-NI-NG-NG
	TalGP3 SL1017	SL1017_7	14.5	NI-NI-NG-HD-NG-NG-HD-NI-NS-HD-NS-HD-NI-NG-NG
	alGR4 SL1017(pseud	SL1017_8	12.5	NS-HD-NS-NI-NG-NI-NI-HD-NI-HD-NI-NG-NG
	TalGO5 SL1017	SL1017_9	6.5	NI-HD-NS-NI-HD-NS-NS
		SL1017_10	4.5	NI-HD-NS-NS-NI
		SL1017_11	1.5	HD-HD
		SL1017_12	0.5	NG
SL1018	TalGN3 SL1018	SL1018-1	34.5	NI-HD-NS-NS-NI-NS-NS-HD-NS-NS-NS-HD-HD-HD-NG-NI-NS-HD-HD-NS-HD-HD-NS-NS-NS-NS-HD-HD-HD-NG-NI-NS-HD-HD-NG
	TalGM3 SL1018	SL1018-2	19.5	NI-NS-HD-NG-HD-NG-NG-HD-NI-NG-NI-NI-HD-NI-HD-NI-HD-NI-NG-NG
	TalGM4 SL1018	SL1018-3	19.5	NI-NS-HD-NG-HD-NG-NG-HD-NI-NG-NI-NI-HD-NI-HD-NI-HD-NI-NG-NG
	TalGQ3 SL1018	SL1018-4	17.5	NI-NS-HD-NI-HD-NG-HD-NG-HD-NI-NG-NS-HD-NI-HD-NI-NG-NG
SL1157	TalGN3 SL1157	SL1157_1	22.5	NI-HD-NS-NS-NI-NS-NS-HD-NS-NS-NS-HD-HD-HD-NG-NI-NS-HD-HD-NS-HD-HD-NG
	TalGM3 SL1157	SL1157_2	19.5	NI-NS-HD-NG-HD-NG-NG-HD-NI-NG-NI-NI-HD-NI-HD-NI-HD-NI-NG-NG
	TalGR3 SL1157	SL1157_3	18.5	NI-HD-NS-NS-NI-NS-NS-HD-NS-NI-NG-NI-NI-HD-NI-HD-NI-NG-NG
	TalGO3 SL1157	SL1157_4	17.5	NI-HD-NS-NI-HD-NS-NS-NS-NS-HD-HD-HD-NG-NI-NS-HD-HD-NG
	TalGQ3 SL1157	SL1157_5	17.5	NI-NS-HD-NI-HD-NG-HD-NG-HD-NI-NG-NS-HD-NS-HD-NI-NG-NG
	TalGP3 SL1157	SL1157_6	14.5	NI-NI-NG-HD-NG-NG-HD-NI-NI-HD-NS-HD-NI-NG-NG
		SL1157_7	14.5	NI-NS-NS-HD-NS-NI-NG-NI-NI-HD-NI-HD-NI-NG-NG
		SL1157_8	0.5	NI
		SL1157_9		
SL1045	lGM3 SL1045 (pseud	1045_1	19.5	NI-NS-HD-NG-HD-NG-NG-HD-NI-NG-NI-NI-HD-NI-HD-NI-HD-NI-NG-NG
	TalGR3 SL1045	1045_2	18.5	NI-HD-NS-NS-NI-NI-NS-HD-NS-NI-NG-NI-NI-HD-NI-HD-NI-NG-NG
	lGQ3 SL1045 (pseud	1045_3	17.5	NI-NS-HD-NI-HD-NG-HD-NG-HD-NI-NG-NS-HD-NS-HD-NI-NG-NG
	TalGP3 SL1045	1045_4	14.5	NI-NI-NG-HD-NG-NG-HD-NI-NS-HD-NS-HD-NI-NG-NG
		1045_5	12.5	HD-NI-NG-NI-NI-HD-NI-HD-NI-HD-NI-NG-NG
		1045_6	9.5	NI-HD-NS-NS-NI-NI-NS-HD-NS-N*
		1045_7	6	NI-NS-HD-NG-HD-NG
		1045_8	5	NI-HD-NS-NS-NI
		1045_9	3.5	HD-NI-NG-NG
		1045_10	2.5	NI-HD-N*
		1045_11	2.5	HD-HD-NG
		1045_12	0.5	N*
		1045_13	0.5	NS
		1045_14		
		1045_15		
		1045_16		

[1] RVDs in red indicate TALEs that are predicted to be encoded on plasmids.

Supplementary Table S2. Clustering of the RVDs from all of the TALEs in Table S1 into six groups based on sequence similarity using the Geneious Alignment program[1,2].

TALE group	AnnoTALE name	No. of Repeats	RVDs (the 12th and 13th amino acid residues of each repeat region)
2	TalGN3 8ra	22.5	NI-HD-NS-NS-NI-NS-NS-HD-NS-NS-NS-HD-HD-HD-NG-NI-NS-HD-HD-NG-HD-HD-NG
	TalGN3 K2	22.5	NI-HD-NS-NS-NI-NS-NS-HD-NS-NS-NS-HD-HD-HD-NG-NI-NS-HD-HD-NG-HD-HD-NG
	TalGN3 SL1017	22.5	NI-HD-NS-NS-NI-NS-NS-HD-NS-NS-NS-HD-HD-HD-NG-NI-NS-HD-HD-NS-HD-HD-NG
	TalGN3 SL1018	34.5	NI-HD-NS-NS-NI-NS-NS-HD-NS-NS-NS-HD-HD-HD-NG-NI-NS-HD-HD-NS-HD-HD-NS-NS-NS-NS-HD-HD-HD-NG-NI-NS-HD-HD-NG
	TalGN3 SL1157	22.5	NI-HD-NS-NS-NI-NS-NS-HD-NS-NS-NS-HD-HD-HD-NG-NI-NS-HD-HD-NS-HD-HD-NG
6	TalGM3 8ra	19.5	NI-NS-HD-NG-HD-NG-NG-HD-NI-NG-NI-NI-HD-NI-HD-NI-HD-NI-NG-NG
	TalGM4 8ra	19.5	NI-NS-HD-NG-HD-NG-NG-HD-NI-NG-NI-NI-HD-NI-HD-NI-HD-NI-NG-NG
	TalGM3 SL1017	19.5	NI-NS-HD-NG-HD-NG-NG-HD-NI-NG-NI-NI-HD-NI-HD-NI-HD-NI-NG-NG
	TalGM3 SL1018	19.5	NI-NS-HD-NG-HD-NG-NG-HD-NI-NG-NI-NI-HD-NI-HD-NI-HD-NI-NG-NG
	TalGM4 SL1018	19.5	NI-NS-HD-NG-HD-NG-NG-HD-NI-NG-NI-NI-HD-NI-HD-NI-HD-NI-NG-NG
	TalGM3 SL1157	19.5	NI-NS-HD-NG-HD-NG-NG-HD-NI-NG-NI-NI-HD-NI-HD-NI-HD-NI-NG-NG
	TalGM3 SL1045	19.5	NI-NS-HD-NG-HD-NG-NG-HD-NI-NG-NI-NI-HD-NI-HD-NI-HD-NI-NG-NG
	K2_6	7	NI-NI-HD-NI-HD-NI-HD
	1045_5	12.5	HD-NI-NG-NI-NI-HD-NI-HD-NI-HD-NI-NG-NG
	1045_7	6	NI-NS-HD-NG-HD-NG
1	TalGR3 8ra	18.5	NI-HD-NS-NS-NI-NS-NS-HD-NS-NI-NG-NI-NI-HD-NI-HD-NI-NG-NG
	TalGR3 K2	18.5	NI-HD-NS-NS-NI-NS-NS-HD-NS-NI-NG-NI-NI-HD-NI-HD-NI-NG-NG
	TalGR3 SL1017	18.5	NI-HD-NS-NS-NI-NS-NS-HD-NS-NI-NG-NI-NI-HD-NI-HD-NI-NG-NG
	TalGR3 SL1157	18.5	NI-HD-NS-NS-NI-NS-NS-HD-NS-NI-NG-NI-NI-HD-NI-HD-NI-NG-NG
	TalGR3 SL1045	18.5	NI-HD-NS-NS-NI-NI-NS-HD-NS-NI-NG-NI-NI-HD-NI-HD-NI-NG-NG
	TalGR4 SL1017	12.5	NS-HD-NS-NI-NG-NI-NI-HD-NI-HD-NI-NG-NG
	SL1017_10	4.5	NI-HD-NS-NS-NI
	SL1157_7	14.5	NI-NS-NS-HD-NS-NI-NG-NI-NI-HD-NI-HD-NI-NG-NG
	1045_6	9.5	NI-HD-NS-NS-NI-NI-NS-HD-NS-N*
	1045_9	3.5	HD-NI-NG-NG
3	TalGO3 8ra	17.5	NI-HD-NS-NI-HD-NS-NS-NS-NS-HD-HD-HD-NG-NI-NS-HD-HD-NG
	TalGO3 K2	17.5	NI-HD-NS-NI-HD-NS-NS-NS-NS-HD-HD-HD-NG-NI-NS-HD-HD-NG
	TalGO3 SL1017	17.5	NI-HD-NS-NI-HD-NS-NS-NS-NS-HD-HD-HD-NG-NI-NS-HD-HD-NG
	TalGO3 SL1157	17.5	NI-HD-NS-NI-HD-NS-NS-NS-NS-HD-HD-HD-NG-NI-NS-HD-HD-NG
	TalGO4 SL1017	17.5	NI-HD-NS-NI-HD-NS-NS-NS-NS-HD-HD-HD-NG-NI-NS-HD-HD-NG
	TalGO5 SL1017	6.5	NI-HD-NS-NI-HD-NS-NS
	K2_7	3.5	NI-NS-HD- -NG
	SL1017_11	1.5	HD-HD
	1045_11	2.5	HD-HD-NG
4	TalGQ3 8ra	17.5	NI-NS-HD-NI-HD-NG-HD-NG-HD-NI-NG-NS-HD-NS-HD-NI-NG-NG
	TalGQ3 K2	17.5	NI-NS-HD-NI-HD-NG-HD-NG-HD-NI-NG-NS-HD-NS-HD-NI-NG-NG
	TalGQ3 SL1018	17.5	NI-NS-HD-NI-HD-NG-HD-NG-HD-NI-NG-NS-HD-NI-HD-NI-NG-NG
	TalGQ3 SL1157	17.5	NI-NS-HD-NI-HD-NG-HD-NG-HD-NI-NG-NS-HD-NS-HD-NI-NG-NG
	TalGQ3 SL1045	17.5	NI-NS-HD-NI-HD-NG-HD-NG-HD-NI-NG-NS-HD-NS-HD-NI-NG-NG
	TalHQ1 SL1017	16.5	NI-NS-HD-NI-HD-NG-HD-NG-HD- -NG-NS-HD-NI-HD-NI-NG-NG
	TalED3 8ra	4.5	NI-HD-NI-NG-NG
5	TalGP3 8ra	14.5	NI-NI-NG-HD-NG-NG-HD-NI-NS-HD-NS-HD-NI-NG-NG
	TalGP3 K2	14.5	NI-NI-NG-HD-NG-NG-HD-NI-NS-HD-NS-HD-NI-NG-NG
	TalGP3 SL1017	14.5	NI-NI-NG-HD-NG-NG-HD-NI-NS-HD-NS-HD-NI-NG-NG
	TalGP3 SL1157	14.5	NI-NI-NG-HD-NG-NG-HD-NI-NI-HD-NS-HD-NI-NG-NG
	TalGP3 SL1045	14.5	NI-NI-NG-HD-NG-NG-HD-NI-NS-HD-NS-HD-NI-NG-NG

[1]The TALE groups are defined based on the clustering shown in Figure 7, with the addition of shorter and potentially incomplete TALEs from Table S1.

[2] RVDs in blue indicate incomplete TALEs; RVDs in red indicate residues showing differences within the same group.

Supplementary Table S3. RVDs of *X. citri* pv. *glycine* TALEs found on plasmids

strain name	TALE group	AnnoTALE name	No. of Repeats	RVDs (the 12th and 13th amino acid residues of each repeat region)
8ra P1 (plasmid)	6	TalGM3 8ra	19.5	NI-NS-HD-NG-HD-NG-NG-HD-NI-NG-NI-NI-HD-NI-HD-NI-HD-NI-NG-NG
	6	TalGM4 8ra	19.5	NI-NS-HD-NG-HD-NG-NG-HD-NI-NG-NI-NI-HD-NI-HD-NI-HD-NI-NG-NG
	4	TalED3 8ra	4.5	NI-HD-NI-NG-NG
SL1017 P1 (plasmid)	1	TalGR3 SL1017	18.5	NI-HD-NS-NS-NI-NS-NS-HD-NS-NI-NG-NI-NI-HD-NI-HD-NI-NG-NG
	3	TalGO4 SL1017	17.5	NI-HD-NS-NI-HD-NS-NS-NS-NS-HD-HD-HD-NG-NI-NS-HD-HD-NG
SL1017 P2	6	TalGM3 SL1017	19.5	NI-NS-HD-NG-HD-NG-NG-HD-NI-NG-NI-NI-HD-NI-HD-NI-HD-NI-NG-NG
SL1018 P1 (plasmid)	6	TalGM3 SL1018	19.5	NI-NS-HD-NG-HD-NG-NG-HD-NI-NG-NI-NI-HD-NI-HD-NI-HD-NI-NG-NG
	6	TalGM4 SL1018	19.5	NI-NS-HD-NG-HD-NG-NG-HD-NI-NG-NI-NI-HD-NI-HD-NI-HD-NI-NG-NG
SL1157 P1 (plasmid)	6	TalGM3 SL1157	19.5	NI-NS-HD-NG-HD-NG-NG-HD-NI-NG-NI-NI-HD-NI-HD-NI-HD-NI-NG-NG
	1	TalGR3 SL1157	18.5	NI-HD-NS-NS-NI-NS-NS-HD-NS-NI-NG-NI-NI-HD-NI-HD-NI-NG-NG
SL1045 P1 (plasmid)	6	TalGM3 SL1045	19.5	NI-NS-HD-NG-HD-NG-NG-HD-NI-NG-NI-NI-HD-NI-HD-NI-HD-NI-NG-NG
